# Supplementary material for: Non-invasive in vivo hyperspectral imaging of the retina for potential biomarker use in Alzheimer’s disease
Source: Nat Commun. 2019 Sep 17;10:4227. doi: 10.1038/s41467-019-12242-1 (PMC6748929; doi:10.1038/s41467-019-12242-1)
Supplement: Supplementary file 1 — Supplementary Information [file 41467_2019_12242_MOESM1_ESM.pdf]

Supplementary Information

Non-invasive in vivo hyperspectral imaging of the retina for potential biomarker use in Alzheimer's disease

Hadoux et al.

Supplementary Methods

Dimension Reduction by Orthogonal Projection (DROP-D)

Considering a matrix **X** of size ($N \times P$), where N is the number of training spectra and P the number of wavelengths in the digitized spectrum and the corresponding matrix **Y** of size ($N \times 2$), where each row codes the class membership (2 classes: PET- control and PET+ case), e.g., [1, 0] corresponds to the first class and [0, 1] correspond to the second class. The matrix **X** is assumed to be centered using the mean spectrum of the data set $\mathbf{x_m}$ of size ($1 \times P$).

The cleaned version of **X** is obtained by computing the projection of **X** orthogonal to the 2 principal components of the within-class covariance matrix as defined by:

$$\mathbf{X_{clean}} = \mathbf{P}^{\perp}_{\mathbf{E_2(W(X,Y))}}\{\mathbf{X}\} \tag{1}$$

Where $\mathbf{W(X,Y) = X^tX - X^tY(Y^tY)^{-1}Y^tX}$ is the within class dispersion matrix of **X** and **Y**, $\mathbf{P^{\perp}_A\{B\} = B(I - A(A^tA)^{-1}A^t)}$ is the orthogonal projection of a matrix $\mathbf{B}$ onto a matrix $\mathbf{A}$, $\mathbf{I}$ the identity matrix of dimension P, and $\mathbf{E_k(A)}$ is a matrix containing the k eigenvectors of $\mathbf{A}$ associated to its k largest eigenvalues.

The spectral model **m**, a vector of dimension (1×P), is derived by extracting the principal axis of the between class covariance matrix as defined by:

$$\mathbf{m} = \mathbf{E_1}\big(\mathbf{B}(\mathbf{X_{clean}}, \mathbf{Y})\big)^t \tag{2}$$

where $\boldsymbol{B(X,Y) = X^tY(Y^tY)^{-1}Y^tX}$ is the between class dispersion matrix of **X** and **Y**.

The HS score (single value) for a new measured spectrum $\mathbf{x_{new}}$ is derived as:

$$\mathbf{s = (x_{new} - x_m)m^t} \tag{3}$$

Determining the selectivity of the spectral model to Aβ

Spectrophotometry of human A$\beta_{1\text{-}42}$

Recombinant human A$\beta_{1\text{-}42}$ protein (Abcam Ab82795, Cambridge, MA, USA) was reconstituted with ultrapure water to concentrations of 1 mg ml^{-1}. The optical density of each peptide solution was measured in triplicate by spectrophotometry and averaged for further analysis (Tecan Sark 20M, Tecan Trading AG, Switzerland) using 200 μl of solution.

Supplementary Discussion

Simulations indicate that the spectral model may be influenced by Aβ

Having demonstrated that the spectral model could be used to reliably distinguish Aβ PET+ cases from PET- controls, we next sought to ascertain whether this spectral difference could be attributed at least in part to a spectral profile similar to that of Aβ. In the absence of validated in vivo measures of the Aβ spectral profile, we performed spectrophotometry of human A$\beta_{1\text{-}42}$ in solution (Supplementary Figure 12). As expected, there was a wavelength-dependent change in the optical density of human A$\beta_{1\text{-}42}$ in solution in the range between 450 – 900 nm. This is typical of Rayleigh-type scattering, affecting shorter wavelengths more than longer wavelengths.

A multiple linear regression was calculated to predict the model based on the spectral profile of haemoglobin (95% saturation), melanin, macular pigment, ocular media (lens) and Aβ (Supplementary Figure 13A). A significant regression equation was found ($F(5, 85) = 173.5$, $p = 4.8e\text{-}43$), with an R^2 of 0.91 and a RMSE of 3.2%. All spectral profiles aside from macular pigment were significant predictors of the model (Supplementary Table 8).

To ensure that the spectral profile of Aβ would not be a significant predictor in the absence of a difference in brain Aβ load between participants we performed another simulation. The spectral data of all participants (Aβ PET+ and PET-) were combined and ranked according to their respective PET Aβ load. Every second spectrum was attributed to class A and the others to class B. In doing so, we ensured that the difference in Aβ between class A and B was minimal. A DROP-D model was then calculated using spectral data from class A and class B. The resulting "simulated" spectral model is presented in Supplementary Figure 13B. A multiple regression was calculated to predict this simulated spectral model based on the spectral profile of haemoglobin (95% saturation), melanin, macular pigment, ocular media (lens) and Aβ. A significant regression equation was found ($F(5, 85) = 240.8$, $p = 1.3e-48$), with an R^2 of 0.93 and a RMSE of 2.8%. As expected, all spectral profiles with the exception of Aβ were significant predictors of this new model (Supplementary Table 9). The Aβ spectral profile is therefore only useful for predicting the shape of the spectral model in the presence of a difference in brain Aβ load.

Supplementary Figures

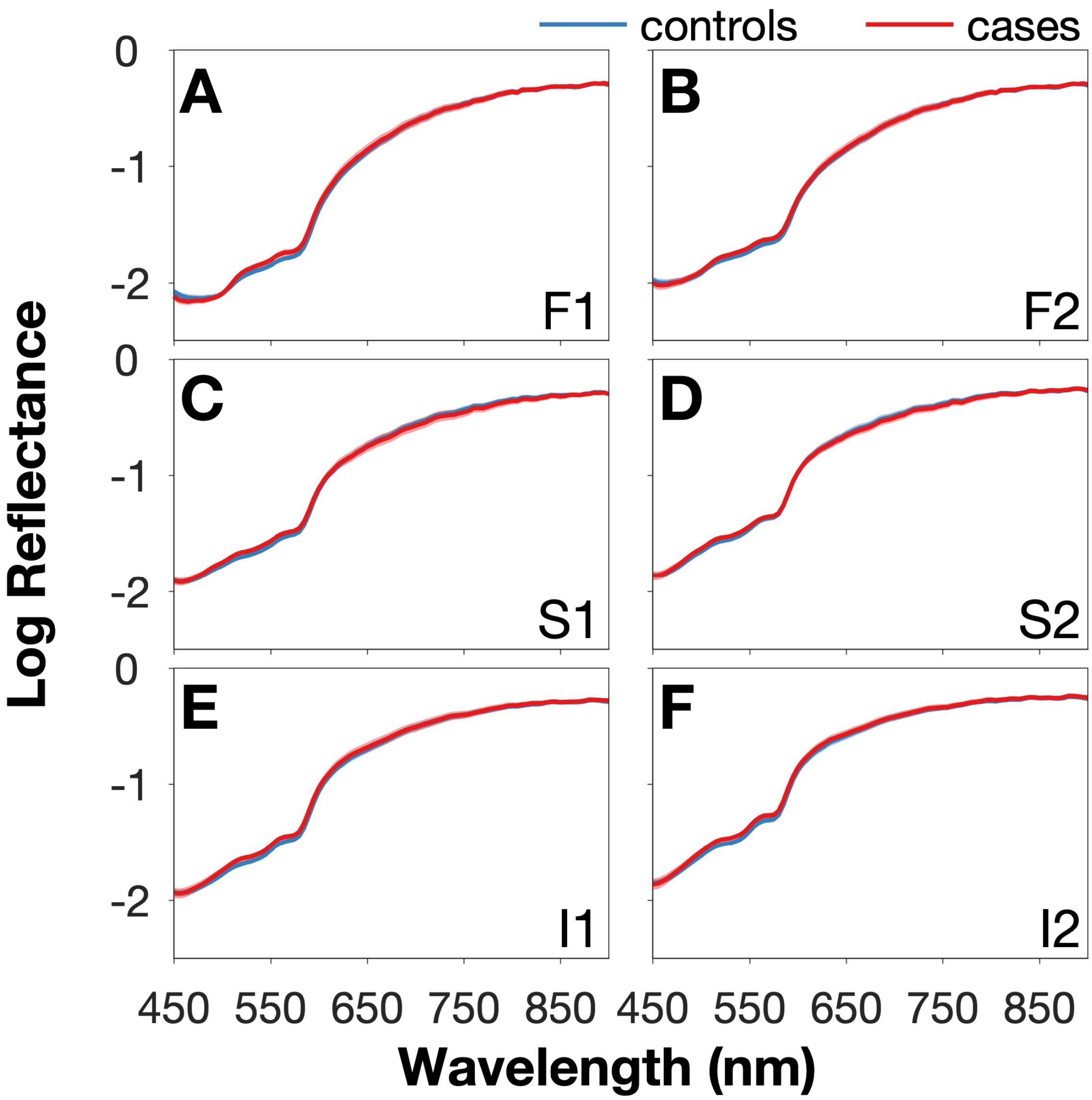

Supplementary Figure 1. Raw reflectance spectra for each sampling location. Spectral data colour-coded by PET status, controls (n = 20, blue) and cases (n = 15, red). **(A, B)** Foveal locations (F1, F2). **(C, D)** Locations superior to the temporal vascular arcades (S1, S2). **(E, F)** Locations inferior to the temporal vascular arcades (I1, I2). The spectral shapes observed in each panel correspond to typical fundus reflectance spectra, i.e., low reflectance in the short wavelength

range which gradually increases (~2 orders of magnitude) with increasing wavelength. The large dynamic range prevents visual comparison between groups. Data shown as mean $\pm$ SEM.

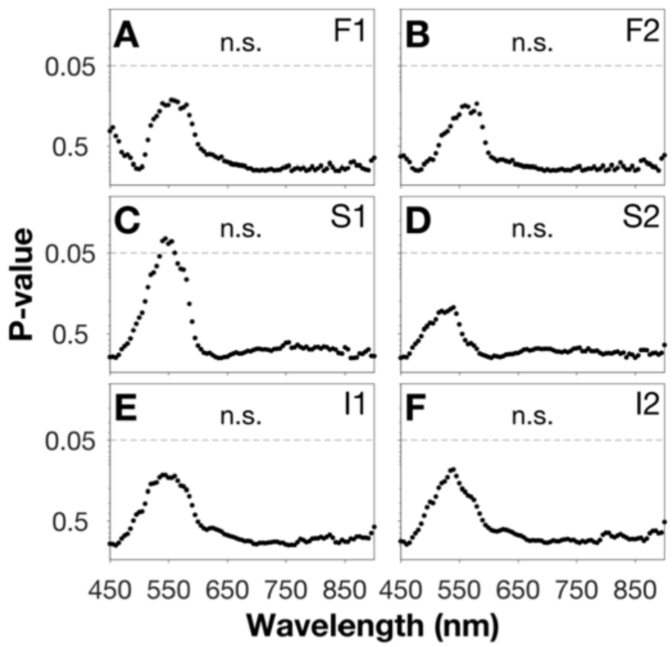

Supplementary Figure 2. No group difference is apparent with raw reflectance data. Controls (n = 20) and cases (n = 15) did not exhibit any significant differences across sampling locations and wavelengths with the raw reflectance data. **(A, B)** Foveal locations (F1, F2). **(C, D)** Locations superior to the temporal vascular arcades (S1, S2). **(E, F)** Locations inferior to the temporal vascular arcades (I1, I2). p-values for two-sided unpaired t-tests between groups are presented for each wavelength. No significant difference was found after controlling for false discovery rate (FDR) for multiple comparisons across all the wavelengths.

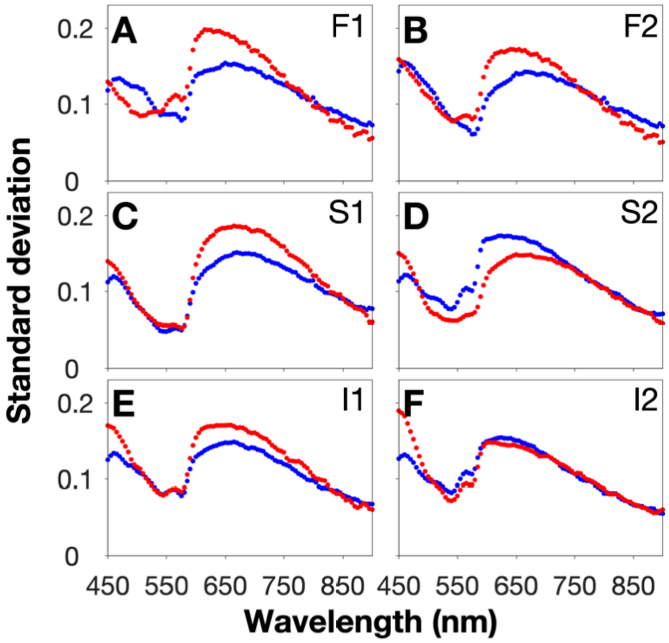

Supplementary Figure 3. Spectral variability is wavelength dependent. The spectral variability of the raw reflectance expressed as the standard deviation is wavelength dependent, but similar across sampling locations for both controls (n = 20, blue) and cases (n = 15, red). **(A, B)** Foveal locations (F1, F2). **(C, D)** Locations superior to the temporal vascular arcades (S1, S2). **(E, F)** Locations inferior to the temporal vascular arcades (I1, I2). All curves show a lower spectral variability at wavelengths centered at 550 nm and above 750 nm.

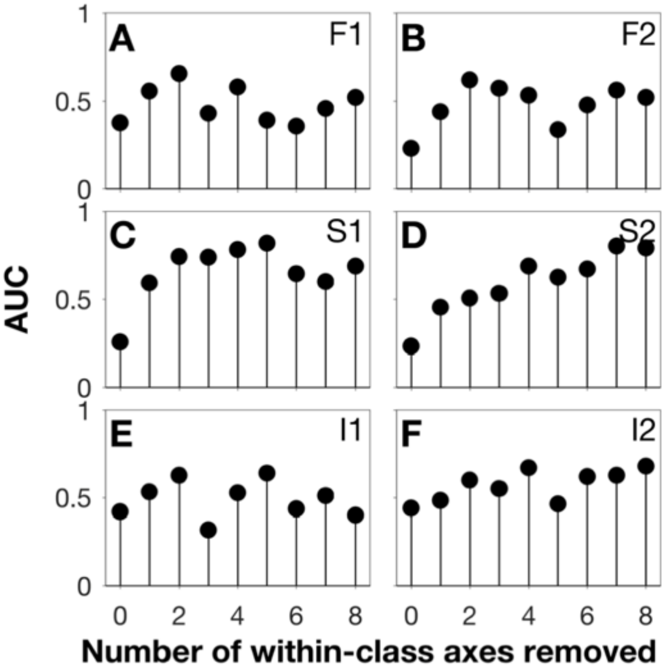

Supplementary Figure 4. Two principal sources of ocular variability prevent discrimination between groups. The single parameter of the DROP-D spectral analysis method (i.e., number of within-class covariance principal axes) was tuned using a leave-one-out cross-validation on the data from the study eye of the principal cohort. Data shows the discrimination performance at each sampling location as more within-class covariance principal axes are removed **(A, B)** Foveal locations (F1, F2). **(C, D)** Locations superior to the temporal vascular arcades (S1, S2). **(E, F)** Locations inferior to the temporal vascular arcades (I1, I2). Discrimination performance was summarised using the area under the receiver operating characteristic (ROC) curve (AUC). Removing 2 axes is the optimal trade-off between model complexity and discrimination performance between cases (n = 15) and controls (n = 20). The optimal number of axes was selected to be at the overall maximum across each sampling location. Selecting more (or fewer) axes would decrease the classification results on average across all sampling locations.

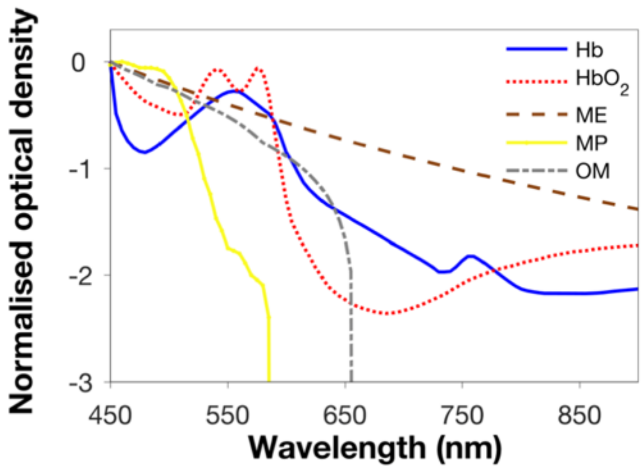

Supplementary Figure 5. Principal ocular sources of spectral variability. Deoxygenated (blue) and oxygenated haemoglobin (red)[1], melanin (brown) [2], macular pigment (yellow) [3] and ocular media (grey) [4]. Spectra are normalised to their largest intensity.

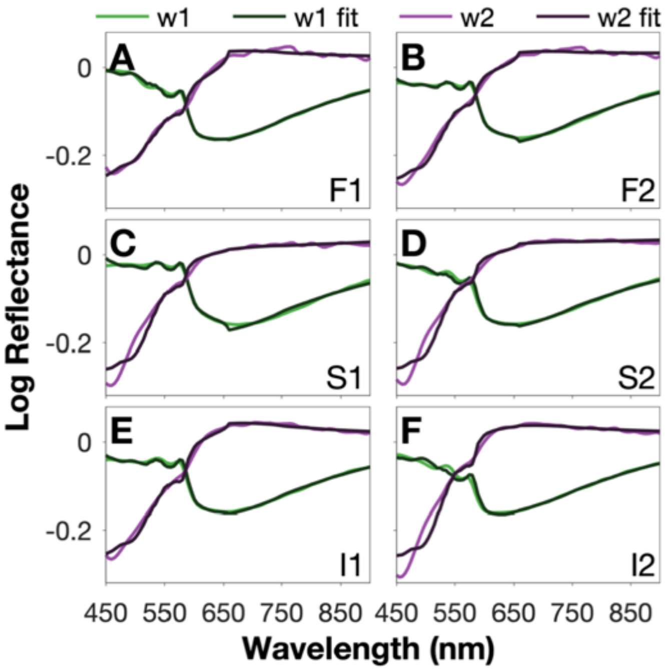

Supplementary Figure 6. Fitting the removed axes to the principal sources of ocular spectral variability. Fitting was performed using multiple linear regression between the spectra of the ocular media, macular pigment, melanin, haemoglobin demonstrating that the removed axes are largely comprised of combinations of these ocular sources (Supplementary Figure 5). Figure panels show fitting for each retinal sampling location: **(A, B)** Foveal locations (F1, F2). **(C, D)** Locations superior to the temporal vascular arcades (S1, S2). **(E, F)** Locations inferior to the temporal vascular arcades (I1, I2). Fitting coefficients are presented for each retinal sampling location in Supplementary Tables 1 to 6.

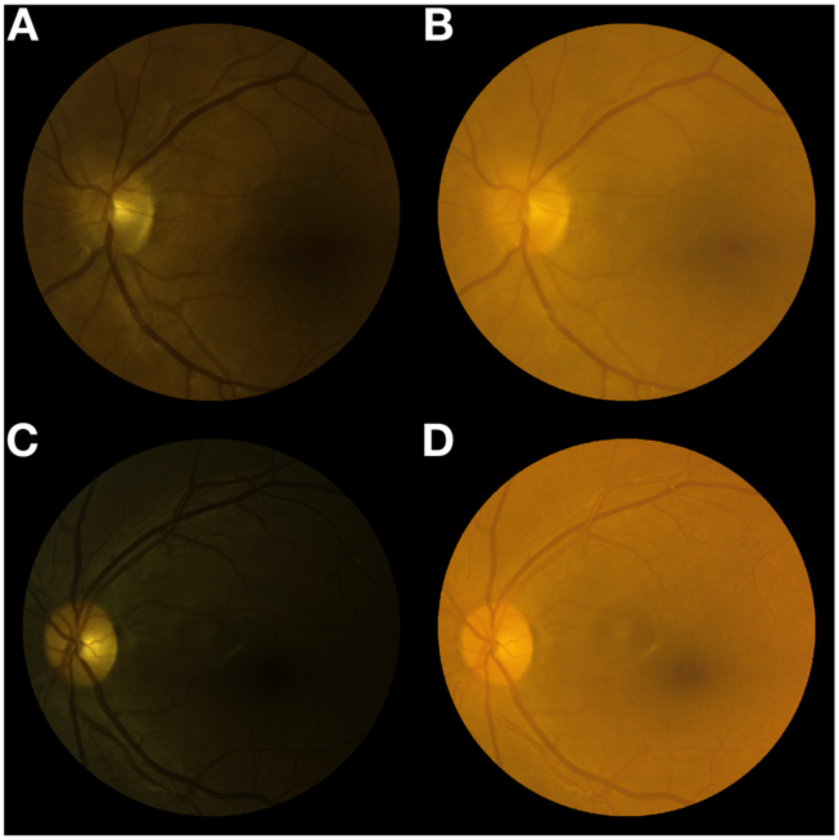

Supplementary Figure 7. Visualisation of the effect of spectral correction using colour reconstruction of fundus images derived from HS images of two participants with marked fundus pigmentation differences. Representative retinal images of a participant with a lightly pigmented fundus **(A)** before and **(B)** after spectral correction. Representative retinal images of a participant with a more heavily pigmented fundus **(C)** before and **(D)** after spectral correction. The variability between fundus images before correction **(A&C)** is reduced once spectral correction is applied **(B&D)**.

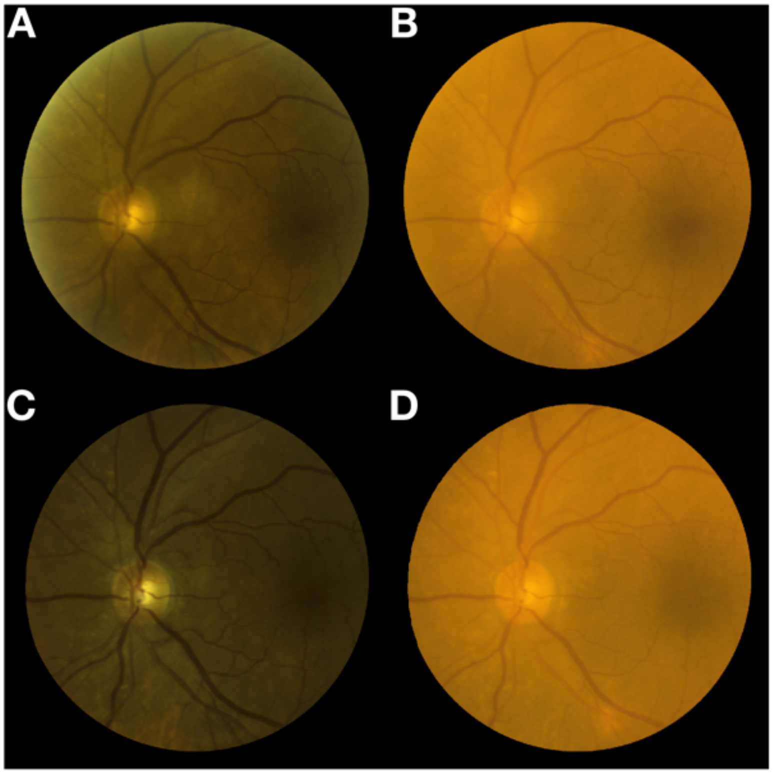

Supplementary Figure 8. Visualisation of the effect of spectral correction using colour reconstruction of fundus images derived from HS images of a participant pre- and post-cataract surgery. Representative retinal images of a participant prior to cataract surgery **(A)** before and **(B)** after spectral correction. Representative retinal images of a participant following cataract surgery **(C)** before and **(D)** after spectral correction. The spectral (colour) difference between fundus images before correction **(A&C)** is reduced once spectral correction is applied **(B&D)**.

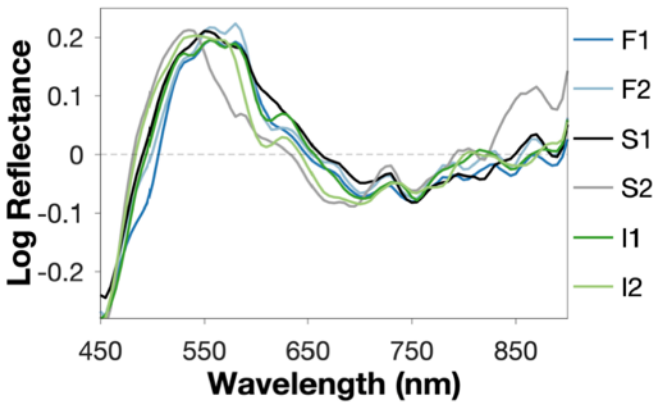

Supplementary Figure 9. Spectral model derived for each sampling location. Spectral models for each sampling location show good agreement despite underlying spectral variability. Good agreement is characterised by having local extrema (wavelength and amplitude) which are similar across sampling locations (i.e., 450, 550 and 750 nm).

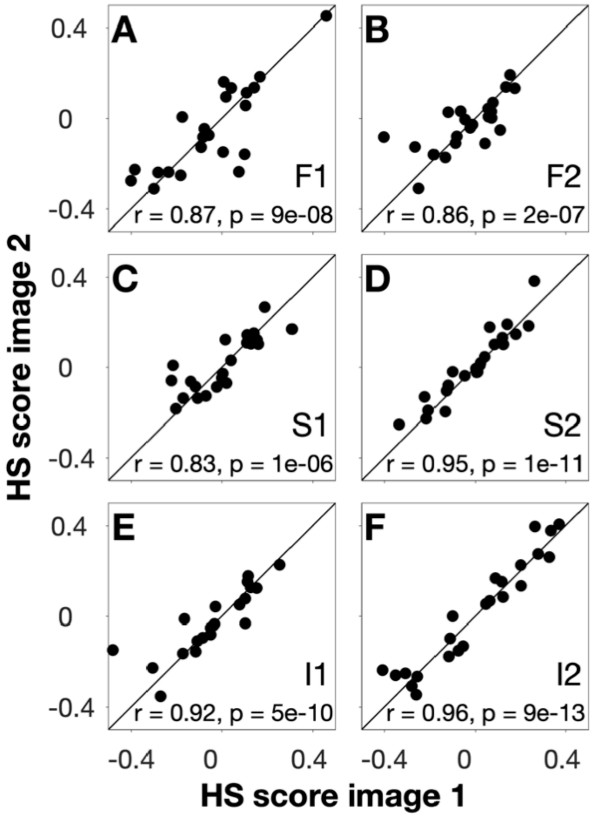

Supplementary Figure 10. Hyperspectral (HS) scores show strong intra-session repeatability across all sampling locations. (A, B) Foveal locations (F1, F2). **(C, D)** Locations superior to the temporal vascular arcades (S1, S2). **(E, F)** Locations inferior to the temporal vascular arcades (I1, I2). HS scores were derived using the spectral model for each location on participants (n = 25) who had two images acquired of the same eye during the same visit.

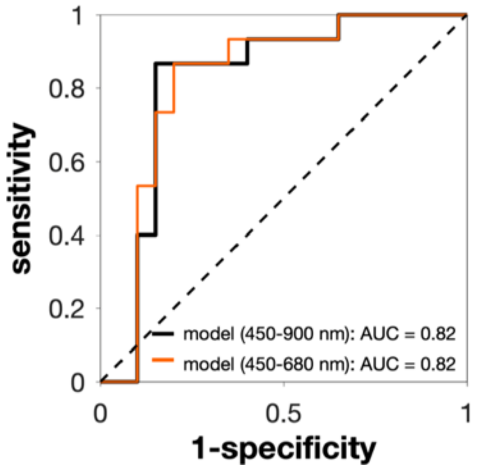

Supplementary Figure 11. The recalibrated spectral model can distinguish between Aβ PET+ and Aβ PET- human participants. When the spectral model was recalibrated for the wavelength range of the mouse imaging system (450 – 680 nm) and the recalibrated model was used to calculate HS scores for the human retinal imaging data, the ability to distinguish Aβ PET+ and PET- participants remained, as indicated by the AUC of the ROC curve.

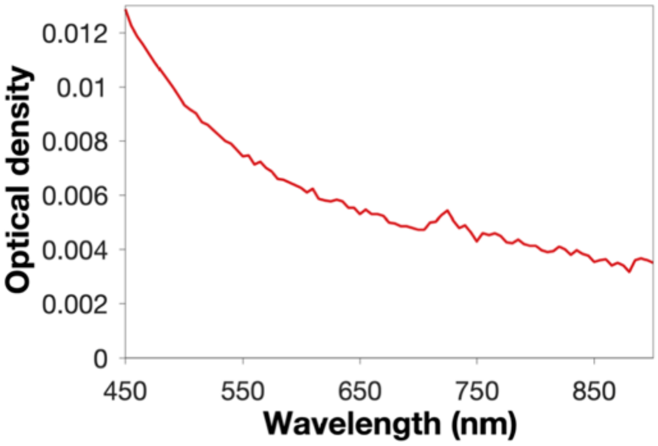

Supplementary Figure 12. Spectrophotometry data for human Aβ1-42 in solution. The optical density of human $A\beta_{1-42}$ in solution (1 mg ml^{-1}) measured by spectrophotometry shows a wavelength dependency that corresponds to loss of light due to Rayleigh-type scattering.

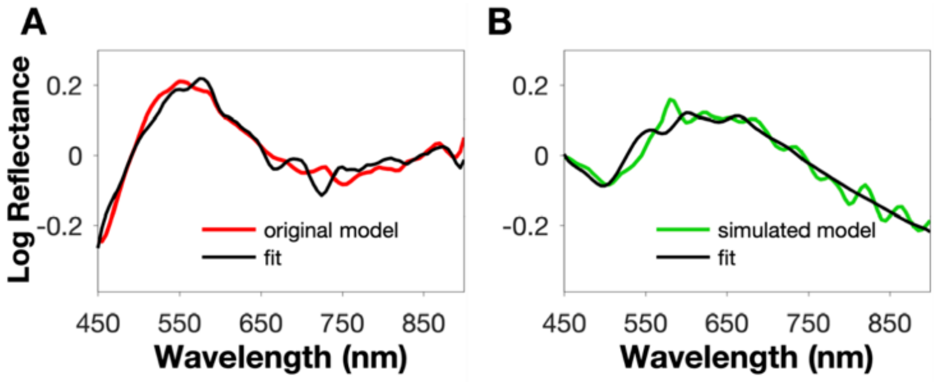

Supplementary Figure 13. Model prediction with multiple linear regression. Multiple linear regression based on the spectral profiles of haemoglobin (95% saturation), melanin, macular pigment, ocular media (lens) and Aβ was used to predict the spectral profile of **(A)** the spectral model of the difference between PET+ and PET- participants and **(B)** the spectral model of the difference between class A and class B participants (participants were allocated into 2 groups, A and B, such that there were no group differences in brain PET Aβ load). The spectral profile of Aβ was only useful for predicting the shape of the spectral model in the presence of a difference in brain Aβ load **(A)** (Supplementary Tables 8 & 9).

Supplementary Table 1. Sampling location F1 – Multiple regression coefficient table. Prediction of w1 and w2 based on the spectral profile of haemoglobin (95% saturation), melanin, macular pigment and ocular media (lens).

	Predictor	Estimate	SE	t-stat	p-value
**w1**	**(Intercept)**	-0.003	0.0009	-3.59	5.5E-04
	**HbO2 (95% Sat)**	0.075	0.0014	53.33	1.1E-67
	**Melanin**	-0.176	0.0030	-58.96	2.5E-71
	**Macular pigment**	0.071	0.0009	79.09	4.2E-82
	**Ocular media**	0.009	0.0021	4.27	5.1E-05
	A significant regression equation was found ($F(4, 86) = 5362.8$, $p = 3.2e-102$), with an R^2 of 0.996 and a RMSE of 0.3%.				
**w2**	**(Intercept)**	-0.249	0.0020	-122.04	3.7E-98
	**HbO2 (95% Sat)**	-0.037	0.0031	-11.87	8.4E-20
	**Melanin**	0.004	0.0065	0.68	5.0E-01
	**Macular pigment**	-0.062	0.0020	-31.41	6.6E-49
	**Ocular media**	-0.050	0.0045	-11.13	2.5E-18
	A significant regression equation was found ($F(4, 86) = 4109.2$, $p = 2.8e-97$), with an R^2 of 0.995 and a RMSE of 0.7%				

Supplementary Table 2. Sampling location F2 – Multiple regression coefficient table. Prediction of w1 and w2 based on the spectral profile of haemoglobin (95% saturation), melanin, macular pigment and ocular media (lens).

	Predictor	Estimate	SE	t-stat	p-value
w1	**(Intercept)**	-0.025	0.0009	-27.08	7.3E-44
	**HbO2 (95% Sat)**	0.082	0.0014	59.62	9.8E-72
	**Melanin**	-0.171	0.0029	-59.02	2.3E-71
	**Macular pigment**	0.039	0.0009	44.51	3.7E-61
	**Ocular media**	0.025	0.0020	12.42	6.9E-21
	A significant regression equation was found ($F(4, 86) = 4764.2$, $p = 5.0e-100$), with an R^2 of 0.996 and a RMSE of 0.3%.				
w2	**(Intercept)**	-0.256	0.0022	-116.12	2.6E-96
	**HbO2 (95% Sat)**	-0.031	0.0033	-9.37	8.7E-15
	**Melanin**	-0.010	0.0071	-1.46	1.5E-01
	**Macular pigment**	-0.082	0.0021	-38.45	5.9E-56
	**Ocular media**	-0.032	0.0049	-6.51	4.9E-09
	A significant regression equation was found ($F(4, 86) = 3634.3$, $p = 5.3e-95$), with an R^2 of 0.994 and a RMSE of 0.8%				

Supplementary Table 3. Sampling location S1 – Multiple regression coefficient table. Prediction of w1 and w2 based on the spectral profile of haemoglobin (95% saturation), melanin, macular pigment and ocular media (lens).

	Predictor	Estimate	SE	t-stat	p-value
**w1**	**(Intercept)**	-0.007	0.0015	-4.46	2.5E-05
	**HbO2 (95% Sat)**	0.082	0.0023	35.71	2.4E-53
	**Melanin**	-0.162	0.0049	-33.27	6.9E-51
	**Macular pigment**	0.034	0.0015	22.97	1.9E-38
	**Ocular media**	0.037	0.0034	10.86	8.4E-18
	A significant regression equation was found (F(4, 86) = 2118.0, p = 5.4e-85), with an R^2 of 0.99 and a RMSE of 0.5%.				
**w2**	**(Intercept)**	-0.262	0.0040	-65.30	4.7E-75
	**HbO2 (95% Sat)**	-0.023	0.0061	-3.86	2.2E-04
	**Melanin**	-0.040	0.0129	-3.12	2.4E-03
	**Macular pigment**	-0.096	0.0039	-24.79	6.2E-41
	**Ocular media**	-0.005	0.0089	-0.61	5.4E-01
	A significant regression equation was found (F(4, 86) = 1024.4, p = 1.2e-71), with an R^2 of 0.98 and a RMSE of 1.4%				

Supplementary Table 4. Sampling location S2 – Multiple regression coefficient table. Prediction of w1 and w2 based on the spectral profile of haemoglobin (95% saturation), melanin, macular pigment and ocular media (lens).

	Predictor	Estimate	SE	t-stat	p-value
**w1**	**(Intercept)**	-0.017	0.0013	-13.49	6.1E-23
	**HbO2 (95% Sat)**	0.072	0.0019	37.10	1.1E-54
	**Melanin**	-0.162	0.0041	-39.43	7.6E-57
	**Macular pigment**	0.057	0.0012	46.40	1.2E-62
	**Ocular media**	0.012	0.0028	4.20	6.4E-05
	A significant regression equation was found ($F(4, 86) = 2295.4$, $p = 1.8e-86$), with an R^2 of 0.99 and a RMSE of 0.5%.				
**w2**	**(Intercept)**	-0.261	0.0040	-65.25	5.0E-75
	**HbO2 (95% Sat)**	-0.022	0.0060	-3.67	4.1E-04
	**Melanin**	-0.024	0.0128	-1.90	6.0E-02
	**Macular pigment**	-0.099	0.0039	-25.77	3.3E-42
	**Ocular media**	-0.015	0.0089	-1.66	1.0E-01
	A significant regression equation was found ($F(4, 86) = 1092.8$, $p = 8.1e-73$), with an R^2 of 0.98 and a RMSE of 1.4%				

Supplementary Table 5. Sampling location I1 – Multiple regression coefficient table. Prediction of w1 and w2 based on the spectral profile of haemoglobin (95% saturation), melanin, macular pigment and ocular media (lens).

	Predictor	Estimate	SE	t-stat	p-value
w1	**(Intercept)**	-0.029	0.0011	-26.47	4.3E-43
	**HbO2 (95% Sat)**	0.081	0.0017	49.22	8.8E-65
	**Melanin**	-0.162	0.0035	-46.19	1.7E-62
	**Macular pigment**	0.043	0.0011	40.68	6.0E-58
	**Ocular media**	0.013	0.0024	5.24	1.2E-06
	A significant regression equation was found (F(4, 86) = 2922.5, p = 5.9e-91), with an R^2 of 0.99 and a RMSE of 0.4%.				
w2	**(Intercept)**	-0.257	0.0021	-123.52	1.3E-98
	**HbO2 (95% Sat)**	-0.047	0.0031	-15.04	8.6E-26
	**Melanin**	0.015	0.0067	2.18	3.2E-02
	**Macular pigment**	-0.088	0.0020	-43.91	1.1E-60
	**Ocular media**	-0.025	0.0046	-5.43	5.0E-07
	A significant regression equation was found (F(4, 86) = 4131.2, p = 2.2e-97), with an R^2 of 0.995 and a RMSE of 0.7%				

Supplementary Table 6. Sampling location I2 – Multiple regression coefficient table. Prediction of w1 and w2 based on the spectral profile of haemoglobin (95% saturation), melanin, macular pigment and ocular media (lens).

	Predictor	Estimate	SE	t-stat	p-value
**w1**	**(Intercept)**	-0.033	0.0017	-20.05	3.6E-34
	**HbO2 (95% Sat)**	0.065	0.0025	25.93	2.1E-42
	**Melanin**	-0.169	0.0053	-31.75	2.9E-49
	**Macular pigment**	0.069	0.0016	43.16	4.6E-60
	**Ocular media**	-0.003	0.0037	-0.73	4.6E-01
	A significant regression equation was found ($F(4, 86) = 1148.9$, $p = 9.8e-74$), with an R^2 of 0.98 and a RMSE of 0.6%.				
**w2**	**(Intercept)**	-0.261	0.0047	-55.35	5.0E-69
	**HbO2 (95% Sat)**	-0.033	0.0071	-4.65	1.2E-05
	**Melanin**	0.010	0.0151	0.65	5.2E-01
	**Macular pigment**	-0.119	0.0046	-26.20	9.3E-43
	**Ocular media**	-0.004	0.0105	-0.41	6.8E-01
	A significant regression equation was found ($F(4, 86) = 806.7$, $p = 2.8e-67$), with an R^2 of 0.97 and a RMSE of 1.7%				

Supplementary Table 7. Cataract participant demographics.

Cataracts are graded in 0.1 increments, in comparison with a set of standardised transparencies which illustrate the integer grades of cataract[5]. Nuclear Opalescence and Nuclear Colour are measures of opacification and brunescence in nuclear sclerotic cataract respectively and are graded on a decimal scale from 1.0 to 6.9. Cortical and Posterior Subcapsular cataract subtypes are graded on a scale from 1.0 to 5.9.

*Continuous variables are expressed as mean ± standard deviation.

†Dichotomous variables are expressed as number of participants in each group.

**Variables**	**Participants (n = 10)**
Age*	68.5 ± 8.1
Sex (Female/Male) †	7/3
**LOCS III grading***	
Nuclear Opalescence*	4.0 ± 1.1
Nuclear Colour*	4.0 ± 1.1
Cortical*	2.6 ± 0.8
Posterior Subcapsular*	0.5 ± 0.8
**Visual acuity (LogMar)**	
Pre-surgery*	0.44 ± 0.22
Post-surgery*	0.15 ± 0.18

Supplementary Table 8. Spectral model (difference between Aβ PET+ and PET- participants) - Multiple regression coefficient table. Prediction of the spectral model based on the spectral profile of haemoglobin (95% saturation), melanin, macular pigment, ocular media (lens) and Aβ.

Predictor	Estimate	SE	t Stat	p-value
**(Intercept)**	1.564	0.1062	14.74	4.0E-25
**HbO2 (95% Sat)**	0.100	0.0137	7.29	1.5E-10
**Melanin**	0.465	0.0473	9.82	1.2E-15
**Macular pigment**	0.026	0.0162	1.59	1.2E-01
**Ocular media**	0.137	0.0201	6.83	1.2E-09
**Aβ**	-1.833	0.1213	-15.11	8.3E-26

Supplementary Table 9. Simulated spectral model (difference between class A and B participants)- Multiple regression coefficient table. Prediction of the simulated spectral model based on the spectral profile of haemoglobin (95% saturation), melanin, macular pigment, ocular media (lens) and Aβ.

Predictor	Estimate	SE	t Stat	p-value
**(Intercept)**	-0.130	0.0914	-1.43	1.6E-01
**HbO2 (95% Sat)**	-0.037	0.0118	-3.12	2.5E-03
**Melanin**	0.570	0.0407	14.00	8.8E-24
**Macular pigment**	-0.197	0.0139	-14.11	5.5E-24
**Ocular media**	-0.082	0.0173	-4.73	9.0E-06
**Aβ**	0.126	0.1044	1.21	2.3E-01

Supplementary References

1. Zonios, G., Bykowski, J. & Kollias, N. Skin melanin, hemoglobin, and light scattering properties can be quantitatively assessed in vivo using diffuse reflectance spectroscopy. *J. Invest. Dermatol.* **117,** 1452–1457 (2001).
2. Goldman, L. The Skin. *Archives of Environmental Health: An International Journal* **18,** 434–436 (1969).
3. Snodderly, D. M., Brown, P. K., Delori, F. C. & Auran, J. D. The macular pigment. I. Absorbance spectra, localization, and discrimination from other yellow pigments in primate retinas. *Investigative Ophthalmology & Visual Science* **25,** 660–673 (1984).
4. Norren, D. V. & Vos, J. J. Spectral transmission of the human ocular media. *Vision Research* **14,** 1237–1244 (1974).
5. Chylack, L. T. *et al.* The Lens Opacities Classification System III. The Longitudinal Study of Cataract Study Group. *Arch Ophthalmol* **111,** 831–836 (1993).
