# Supplementary material for: Non-invasive in vivo hyperspectral imaging of the retina for potential biomarker use in Alzheimer’s disease
Source: Nat Commun. 2019 Sep 17;10:4227. doi: 10.1038/s41467-019-12242-1 (PMC6748929; doi:10.1038/s41467-019-12242-1)
Supplement: Supplementary file 3 — Description of Additional Supplementary Files [file 41467_2019_12242_MOESM3_ESM.pdf]

Description of Additional Supplementary Files

File Name: Supplementary Movie 1
Description: Acquisition of HS image using the MHRC. Real time and slow motion (x4) acquisition showing the illumination sweeping through the wavelengths (900 to 450 nm) in 0.9 seconds. The recorded HS image frames of the fundus are shown in the inset image.
