# Supplementary material for: Non-invasive in vivo hyperspectral imaging of the retina for potential biomarker use in Alzheimer’s disease
Source: Nat Commun. 2019 Sep 17;10:4227. doi: 10.1038/s41467-019-12242-1 (PMC6748929; doi:10.1038/s41467-019-12242-1)
Supplement: Supplementary file 5 — Reporting Summary [file 41467_2019_12242_MOESM5_ESM.pdf]

natureresearch

Corresponding author(s): Xavier Hadoux

Reporting Summary

Nature Research wishes to improve the reproducibility of the work that we publish. This form provides structure for consistency and transparency in reporting. For further information on Nature Research policies, see Authors & Referees and the Editorial Policy Checklist.

Statistical parameters

When statistical analyses are reported, confirm that the following items are present in the relevant location (e.g. figure legend, table legend, main text, or Methods section).

n/a	Confirmed	
☐	☒	The exact sample size (n) for each experimental group/condition, given as a discrete number and unit of measurement
☐	☒	An indication of whether measurements were taken from distinct samples or whether the same sample was measured repeatedly
☐	☒	The statistical test(s) used AND whether they are one- or two-sided<br>*Only common tests should be described solely by name; describe more complex techniques in the Methods section.*
☐	☒	A description of all covariates tested
☐	☒	A description of any assumptions or corrections, such as tests of normality and adjustment for multiple comparisons
☐	☒	A full description of the statistics including central tendency (e.g. means) or other basic estimates (e.g. regression coefficient) AND variation (e.g. standard deviation) or associated estimates of uncertainty (e.g. confidence intervals)
☐	☒	For null hypothesis testing, the test statistic (e.g. F, t, r) with confidence intervals, effect sizes, degrees of freedom and P value noted<br>*Give P values as exact values whenever suitable.*
☒	☐	For Bayesian analysis, information on the choice of priors and Markov chain Monte Carlo settings
☒	☐	For hierarchical and complex designs, identification of the appropriate level for tests and full reporting of outcomes
☐	☒	Estimates of effect sizes (e.g. Cohen's d, Pearson's r), indicating how they were calculated
☐	☒	Clearly defined error bars<br>*State explicitly what error bars represent (e.g. SD, SE, CI)*

Our web collection on statistics for biologists may be useful.

Software and code

Policy information about availability of computer code

Data collection	PhySpec (2.6.0, Photon Etc.) was used to record the hyperspectral images.
Data analysis	All analyses were performed using Matlab (R2017a, The MathWorks Inc., Natick, MA, USA) or Prism (v6.0, GraphPad Software Inc., La Jolla, CA, USA)

For manuscripts utilizing custom algorithms or software that are central to the research but not yet described in published literature, software must be made available to editors/reviewers upon request. We strongly encourage code deposition in a community repository (e.g. GitHub). See the Nature Research guidelines for submitting code & software for further information.

Data

Policy information about availability of data

All manuscripts must include a data availability statement. This statement should provide the following information, where applicable:

- Accession codes, unique identifiers, or web links for publicly available datasets
- A list of figures that have associated raw data
- A description of any restrictions on data availability

Principal cohort clinical data were collected at the Centre for Eye Research Australia in collaboration with the AIBL study. Deidentified clinical data for the validation

set were obtained from Optina Diagnostics. These data are not publicly available, and restrictions apply to their use. Source data of graphs are available as source files upon request.

Field-specific reporting

Please select the best fit for your research. If you are not sure, read the appropriate sections before making your selection.

☒ Life sciences ☐ Behavioural & social sciences ☐ Ecological, evolutionary & environmental sciences

For a reference copy of the document with all sections, see nature.com/authors/policies/ReportingSummary-flat.pdf

Life sciences study design

All studies must disclose on these points even when the disclosure is negative.

Sample size	A sample size of 20 was necessary to detect an area under the receiver operating curve of at least 0.8 with a power of 80% and a significance level of 0.05. The 35 participants recruited in this study is above this requirement.
Data exclusions	Exclusion criteria included age under 30 years; opacification of the ocular media such as visually significant cataract (associated with visual acuity 6/18 or worse) or corneal disease sufficient to preclude retinal imaging; angle closure glaucoma or angle-closure suspects; retinal surgery in the preceding 6 months; previous major ocular trauma; retinal dystrophy and participants with any medical conditions likely to preclude satisfactory retinal imaging.<br><br>Three participants (PET+) were excluded from the study due to posterior capsular opacification after artificial lens implantation that prevented successful imaging. (as per protocol described in the Method section).
Replication	Findings obtained with the images of a single eye of each participants were replicated successfully in their fellow eye. A validation cohort was also imaged according to the same protocol and results were replicated successfully.
Randomization	Participants were allocated in each group based on their PET scan results.
Blinding	In developing the model using data from the principal cohort knowledge of PET status (positive vs negative) alone was known to the investigators. The quantitative PET data were provided (by co-author CJF) after the analytical model was finalised and correlation between HS score and PET quantitative values were performed in a blinded fashion. Blinding was not necessary for cross validation studies (fellow eye study and validation cohort) as parameters of the analytical model were fixed.

Reporting for specific materials, systems and methods

Materials & experimental systems

n/a	Involved in the study
☒	☐ Unique biological materials
☐	☒ Antibodies
☒	☐ Eukaryotic cell lines
☒	☐ Palaeontology
☐	☒ Animals and other organisms
☐	☒ Human research participants

Methods

n/a	Involved in the study
☒	☐ ChIP-seq
☒	☐ Flow cytometry
☒	☐ MRI-based neuroimaging

Antibodies

Antibodies used	Monoclonal mouse antibody 1E8 raised to human Aβ17-24 was used at a dilution ratio of 1:500 to detect total Aβ.
Validation	Culvenor, J. G. et al. Subcellular localization of the Alzheimer's disease amyloid precursor protein and derived polypeptides expressed in a recombinant yeast system. Amyloid 5, 79–89 (1998).<br><br>Tammer, A. H. et al. Generation of a recombinant Fab antibody reactive with the Alzheimer's disease-related Abeta peptide. Clin. Exp. Immunol. 129, 453–463 (2002).

Animals and other organisms

Policy information about studies involving animals; ARRIVE guidelines recommended for reporting animal research

Laboratory animals	The 5xFAD mice colony was established in-house using breeder pairs of 5xFAD mice (MMRRC Stock 34848, Jackson Laboratories, Bar Harbor, ME, USA) and C57BL/6J mice. This strain (B6.CgTg(APPSwFlLon,PSEN1*M146L*L286V)6799Vas/Mmjax) backcrossed on a C57BL/6J background does not carry the retinal degeneration allele Pde6brd1. Mice were genotyped using tail tissue samples shortly after birth by an external vendor (TransnetYX, Cordova, TN, USA). Non-transgenic littermates served as controls.
Wild animals	*Provide details on animals observed in or captured in the field; report species, sex and age where possible. Describe how animals were caught and transported and what happened to captive animals after the study (if killed, explain why and describe method; if released, say where and when) OR state that the study did not involve wild animals.*
Field-collected samples	*For laboratory work with field-collected samples, describe all relevant parameters such as housing, maintenance, temperature, photoperiod and end-of-experiment protocol OR state that the study did not involve samples collected from the field.*

Human research participants

Policy information about studies involving human research participants

Population characteristics	There were no differences between the groups for age and sex (Table 1). On average, controls scored higher in the mini mental state examination (MMSE, $p < 0.0001$) compared to the cases (Table 1). No differences were found between groups for lens status, the presence of macular and peripheral drusen, presence of glaucoma, or retinal nerve fibre layer (RNFL) thickness on the basis of clinical examination, colour fundus photography and optical coherence tomography (Table 1).
Recruitment	Participants in the principal cohort were recruited from two sources, the Royal Melbourne Hospital Neuropsychiatry Unit and the Australian Imaging, Biomarker & Lifestyle Study of Ageing (AIBL) study. All cases had either mild cognitive impairment or early Alzheimer's disease (AD) as determined by a neuropsychiatric test battery and moderate-high A� burden measured on brain PET imaging prior to study commencement. The tracer-specific standardized uptake value ratio (SUVR) diagnostic thresholds for PET positivity were PiB � 1.40, NAV4694 � 1.40, Flutemetamol � 0.55 and Florbetapir � 1.05. The control group included age- and sex-matched participants with amyloid burden on PET scan below the diagnostic threshold for AD, or where A� distribution was atypical for AD.
